# An effective deep learning approach for the classification of Bacteriosis in peach leave

**DOI:** 10.3389/fpls.2022.1064854

**Published:** 2022-11-24

**Authors:** Muneer Akbar, Mohib Ullah, Babar Shah, Rafi Ullah Khan, Tariq Hussain, Farman Ali, Fayadh Alenezi, Ikram Syed, Kyung Sup Kwak

**Affiliations:** ^1^ Institute of Computer Science & Information Technology, The University of Agriculture, Peshawar, Pakistan; ^2^ College of Technological Innovation, Zayed University, Dubai, United Arab Emirates; ^3^ High Performance Computing and Networking Institute, National Research Council (ICAR-CNR), Naples, Italy; ^4^ Department of Software, Sejong University, Seoul, South Korea; ^5^ Department of Electrical Engineering, College of Engineering, Jouf University, Sakaka, Saudi Arabia; ^6^ School of Computing, Gachon University, Seongnam-Si, South Korea; ^7^ Department of Information and Communication Engineering, Inha University, Incheon, South Korea

**Keywords:** peach leaves, Bacteriosis detection, Bacteriosis classification, deep learning, convolutional neural network (CNN), LWNet

## Abstract

Bacteriosis is one of the most prevalent and deadly infections that affect peach crops globally. Timely detection of Bacteriosis disease is essential for lowering pesticide use and preventing crop loss. It takes time and effort to distinguish and detect Bacteriosis or a short hole in a peach leaf. In this paper, we proposed a novel LightWeight (WLNet) Convolutional Neural Network (CNN) model based on Visual Geometry Group (VGG-19) for detecting and classifying images into Bacteriosis and healthy images. Profound knowledge of the proposed model is utilized to detect Bacteriosis in peach leaf images. First, a dataset is developed which consists of 10000 images: 4500 are Bacteriosis and 5500 are healthy images. Second, images are preprocessed using different steps to prepare them for the identification of Bacteriosis and healthy leaves. These preprocessing steps include image resizing, noise removal, image enhancement, background removal, and augmentation techniques, which enhance the performance of leaves classification and help to achieve a decent result. Finally, the proposed LWNet model is trained for leaf classification. The proposed model is compared with four different CNN models: LeNet, Alexnet, VGG-16, and the simple VGG-19 model. The proposed model obtains an accuracy of 99%, which is higher than LeNet, Alexnet, VGG-16, and the simple VGG-19 model. The achieved results indicate that the proposed model is more effective for the detection of Bacteriosis in peach leaf images, in comparison with the existing models.

## 1 Introduction

Plants are an essential aspect of all species’ lives on Earth. The world is an entire green planet as they supply fresh oxygen to breathe and minimize pollution by absorbing carbon dioxide. Plants are directly or indirectly responsible for our food source. They also protect a variety of different creatures. Plants that existed millions of years ago are also used to produce coal, natural gas, and gasoline. Plants are also a valuable source of medication. As a result, a thorough understanding of plants is required to investigate the plant’s genetic link. According to Martinelli et al., 2015 the total and acknowledged plant species number around 373,000, of which 309,312 are vascular plants and 296,383 are flowering plants”. People used their leaves, stems, fruits, flowers, and other parts to identify plants (Behera et al., 2018).

Many countries rely on agricultural products and allied businesses as their primary source of income. One of the most basic and crucial necessities for any country is the safety and security of agricultural products. Pakistan, like other developing countries, has always faced problems of malnourishment. Malnutrition is strongly intertwined with achieving food security (agricultural sector in Pakistan).

There are many different sorts of fruit, but Peach is one of the most popular and liked fruit around the globe due to its taste and other benefits for human health. Peach is high in antioxidants, vitamin C and minerals, including copper, manganese, calcium, magnesium, zinc, phosphorus, iron, and potassium (Krizhevsky et al., 2017). Peach production has led to the financial wealth of developed countries while also significantly influencing the economic development of emerging nations (Otsu, 1979). Peach farming is an essential element of the Pakistani agriculture sector. Peach fruits are popular fruits consumed by millions of people worldwide. Swat region is the top producer of Peach fruit. Swat produces 5280 tons of Peach fruit, and the tribal areas yield 3374, whereas Mardan produces 2825 tons of fruits https://tribune.com.pk/story/2078453/not-peachy-pakistans-peach-growers. Also, the Malakand area yields 1190 tons, followed by Peshawar, which produces 1066 tons of Peaches, according to the crops reporting service of Khyber Pakhtunkhwa in 2018-2019. Moreover, Buner provides 3,105 tons and Upper Dir 1,917 tons. Total production from the tribal district stood at 3,374 tons. This vast yield of Peach fruit plays a crucial part in the economy of Khyber Pakhtunkhwa.

However, several diseases can attack peaches, including Bacterial spots, also known as Bacteriosis or shot holes. Bacteriosis severely affects peach crop production. Older nectarines and peaches are also affected by Bacteriosis infection. Bacteriosis typically develops on the peach leaves first; therefore, the leaves are the primary source for recognizing plant disease (Ebrahimi et al., 2017). The “bacterium Xanthomonas campestris pv. Pruni” causes peach leaf shot-hole diseases. Bacterial spot on the peach fruits induces fruit losses and general tree malaise due to repeated defoliation. This disease can potentially destroy crops across an entire field, resulting in considerable loss of revenue because quality fruit would not be produced from the field (Deepalakshmi et al., 2021).

As a result, early detection of this disease’s infestation is critical to reduce pesticide use, prevent peach fruit loss, and avoid an economic loss to the farmer and country. Early detection of Bacteriosis necessitates a routine professional assessment of the disease’s severity (Martinelli et al., 2015). It is necessary to develop fast and automated methods for classifying Bacteriosis in Peach leaves since manually identifying this disease is labor-intensive and does not produce results well. Image processing is an adequate substitute for automatically identifying diseases from raw leaf pictures. Numerous attempts have been made to identify the images and use a particular classifier to categorize the input leaves images as infected or normal. The following are the contribution of this paper.

- A synthetic dataset is developed which consists of 10000 images: 4500 are Bacteriosis and 5500 are healthy images.
- Novel methods are utilized to accurately preprocess the images for the identification of Bacteriosis and healthy leaves.
- A lightweight LWNet model is proposed to classify peach leaves into infected (Bacteriosis) and healthy with higher accuracy by varying layers and fine-tuning the parameters.
- To evaluate the performance of the proposed LWNet Model, we compare it with the state-of-the-art CNN Models like AlexNet, LeNet, VGG-16, and VGG-19 based on the simulation time, Accuracy, Mean Square Error loss, precision, Recall, and F-Measure.
- The proposed LWNet CNN model improves the performance and achieves a higher accuracy of 99% in the detection of Bacteriosis in peach leaf images, compared to the other four existing models.

The rest of this paper is organized as follows. Section 2 shows the literature review of leaf classification using deep learning methods. Section 3 presents the methodology of the proposed models. Section 4 shows the experimental results. Finally, Section 5 concluded the proposed work.

## 2 Literature review

The authors presented an innovative method for detecting rice and leaf disease based on deep convolutional neural networks (CNNs) Lu et al., 2017 and Shoaib et al., 2022. Various models were trained to detect ten types of rice diseases. They experimented with a dataset consisting of 500 images of healthy and infected rice leaves. Their proposed Model achieved 95.48% accuracy by adopting 10-fold cross-validation. Sethy et al., 2017 introduced the K-Means clustering technique for detecting defective rice leaf parts. They also calculated the affected area. A classification method of SVM with K-means and fuzzy C-means clustering was prepared to recognize the five distinct types of the scarcity of rice crops from leaf pictures and achieved 85 and 90 percent accuracy. Islam et al., 2017 described a procedure for diagnosing diseases from leaf images. This program analyzed a dataset named “Plant Village,” a collection of publicly available datasets to find diseases in potato plants. A segmentation method and an SVM were used to categorize diseases in over 300 images, with a median accuracy of 95%. Oppenheim and Shani, 2017 provided a classification system for potato diseases using computer vision deep learning approaches. The algorithm divides the tubers into five classes, including four infection classes and a healthy potato class. The images in this study contained potatoes of various sizes, shapes, and diseases, which experts meticulously gathered, recognized, and labeled. Dias et al., 2018 proposed a strategy for fine-tuning a pre-trained convolutional neural network to become especially sensitive to flowers presented in this research. The solution surpasses three algorithms representing state-of-the-art flower detection, with recall and accuracy rates of more than 90% based on experimental results on a challenging data set. Liu et al., 2017 used deep convolutional neural networks to detect apple leaf disease. A CNN modal was trained on a data set of 13,689 images of apple leaves to classify apple leaf disease (brown spot, rust, mosaic, and Alternaria leaf spot) into healthy and infected classes. According to experimental findings, the proposed CNNs-based approach gained an overall accuracy of 97.62 percent. When compared to the AlexNet Model, the model parameters are reduced by 51,206,928, and the accuracy of the suggested Model with generated pathological images is improved by 10.83 percent. This study reveals that the suggested deep learning model is more accurate, has a faster convergence rate in detecting apple leaf diseases, and increases the CNNs network modal’s robustness. Behera et al., 2018 worked to detect disease in oranges and classify the sort of flaw. First, a citrus disease review was conducted to create a dataset of digitalized oranges that were categorized by kind of fault and served as a training set. The symptoms of an orange disease show the severity of the disease and might help decide on the best treatment option. To avoid serious harm to oranges yield, it is also vital to diagnose the disease appropriately and promptly. Treatment of orange diseases is more expensive and pollutes the environment due to the overuse of pesticides. As a result, pesticide use was reduced. This research used SVM with numerous classes and k-means clustering, and Fuzzy Logic to calculate the seriousness of orange sickness to classify diseases accurately with a 90% accuracy. Geetharamani and Pandian, 2019 worked on the Deep CNN model using an available dataset containing 39 types of plant leave images. Six data augmentation methods were employed: noise injection, principal component analysis (PCA), Gamma correction, image flipping, color augmentation, rotation, and scaling to make CNN models more effective. The suggested Model functions more effectively when the validation data are used. The suggested Model obtains a classification accuracy of 96.46 percent after extensive simulation. Ozguven and Adem, 2019 worked on an automatic diagnosis of Cercospora beticola Sacc, also known as leaf spot disease, in sugar beet. They used a Faster R-CNN architecture by modifying the parameters of a CNN model on 155 images and succeeded with 95.48% accuracy. Zhang et al., 2019 developed a “13-layer convolutional neural network” for their research (CNNs). Data augmentation techniques used gamma correction, image rotation, and noise injection. compared the maximum and average pooling as well. Using stochastic gradient descent (SGD) with momentum and a minibatch size of 128, the CNNs were trained. The suggested methodology outperforms state-of-the-art methods by at least five percentage points, with a general accuracy of 94.94 percent. It was discovered that data augmentation could improve Accuracy. Alehegn, 2019 attempted to build maize leaf disease recognition and classification using an image processing and support vector machine model. Eight hundred images total, of which 80% were employed for training and 20% for testing, were used to analyze the modal’s acceptance and classification precision. The support vector machine supported the experiment result, which achieved a median accuracy of 95.63 percent using integrated (texture, color, and morphology) information. El-kahlout et al., 2019 used a dataset of 2,306 photos, and a machine learning-based technique for differentiating types of peaches is proposed. 1,212 images were used for training, 520 for validation, and 574 for testing. The researchers used a deep learning method widely used in picture recognition. 30% of the image is used for validation, while the remaining 70% is used for training. Our trained Model produced 100% accuracy on a held-out test set. Ahila Priyadharshini et al., 2019 Proposed a classification architecture for maize leaf disease based on “Deep Convolutional Neural Networks (CNN) (modified LeNet)”. Maize leaf images from the Plant Village dataset are utilized in the studies. The proposed CNNs can distinguish between four separate classes (three diseases and one healthy class). The learned Model has a 97.89 percent accuracy rate. Chen et al., 2020 looked at the deep learning strategy to solve the problem in this study because it has shown to be very good at image processing and classification challenges. The Dense Net module, which was pre-trained in ImageNet, and the Inception module were chosen for usage in the network since they combined both benefits. In comparison to other state-of-the-art methods, this approach outperforms them. The public dataset has an average prediction accuracy of 94.07 percent. The average class prediction accuracy for photographs of rice disease is 98.63 percent, even when several diseases are taken into account. da Costa et al., 2020 developed a deep learning technique to identify exterior problems in this paper. There are 43,843 images in the data collection and external faults. This online dataset indicates a significant imbalance in terms of healthy images. A deep Residual Neural Network (DRN) is a type of neural network that has a (ResNet). A classifier was trained to detect external faults through feature extraction and fine-tuning. The Model had an average precision of 94.6 percent on the test set. & The Optimal Classifier has an 86.6 percent recall while retaining precision of 91.7%. Joshi et al., 2021 introduced an automated deep learning base of viral infection detection for Vigna Mungo L., a leguminous plant mostly produced in the Indian subcontinent. Creating an automatic disease detection approach that can conduct jobs in real time is challenging. Adding variation to the leaf image data collection, the image data set acquired from several kinds of Vigna Mongo leaves split and enhanced. The convolutional neural network VirLeafNe was trained with different leaf images for numerous epochs, including healthy, slightly diseased, and badly infected leaves. Drone sprayers can be used with the proposed methodology to analyze larger crop regions. The suggested method is fully automated, non-destructive, and classifies leaf images into many categories in real-time. After thorough algorithm tests, all proposed models obtained high levels of validation accuracy, with testing accuracy for VirLeafNet1, VirLeafNet2, and VirLeafNet3 of 91.234 percent, 96.429 percent, and 97.403 percent, respectively, on diverse leaf images. Goncharov et al., 2020 described a dataset with healthy and infected leaf images of wheat, corn, and grapes plants. They proposed an architecture that uses a deep Siamese network as a feature extractor and a single-layer perceptron as a classifier, achieving an Accuracy of 96%. Sharma et al., 2020 looked into a possible solution by training convolutional neural network (CNN) models with segmented image data. The S-CNN model beats the F-CNN modal by doubling its performance to 98.6% accuracy compared to the F-CNN modal trained using entire images. This performance was tested on independent data previously unnoticed by the modals. Additionally, we demonstrate that the S-CNN model’s self-classification confidence is significantly greater than the F-CNN Model using a tomato plant and a target spot disease as an example. This study moves the utility of automated disease diagnosis systems closer to laypeople. Yadav et al., 2021 worked on CNN models to detect Bacteriosis in peach leaf images. An adaptive operation was conducted to a chosen suitable color image channel, and the affected region’s disease spots were quantified. To segment and identify bacterial spots, grey-level slicing is applied to pre-processed leaf images. The datasets have been augmented to make the algorithm resistant to varying lighting conditions. Results showed that their Model achieved an accuracy of 98.75 percent and a time of 0.185 seconds per image to detect abacterial and healthy leaves. tool due to its high detection rate.

## 3 Methodology

**Figure 1:**
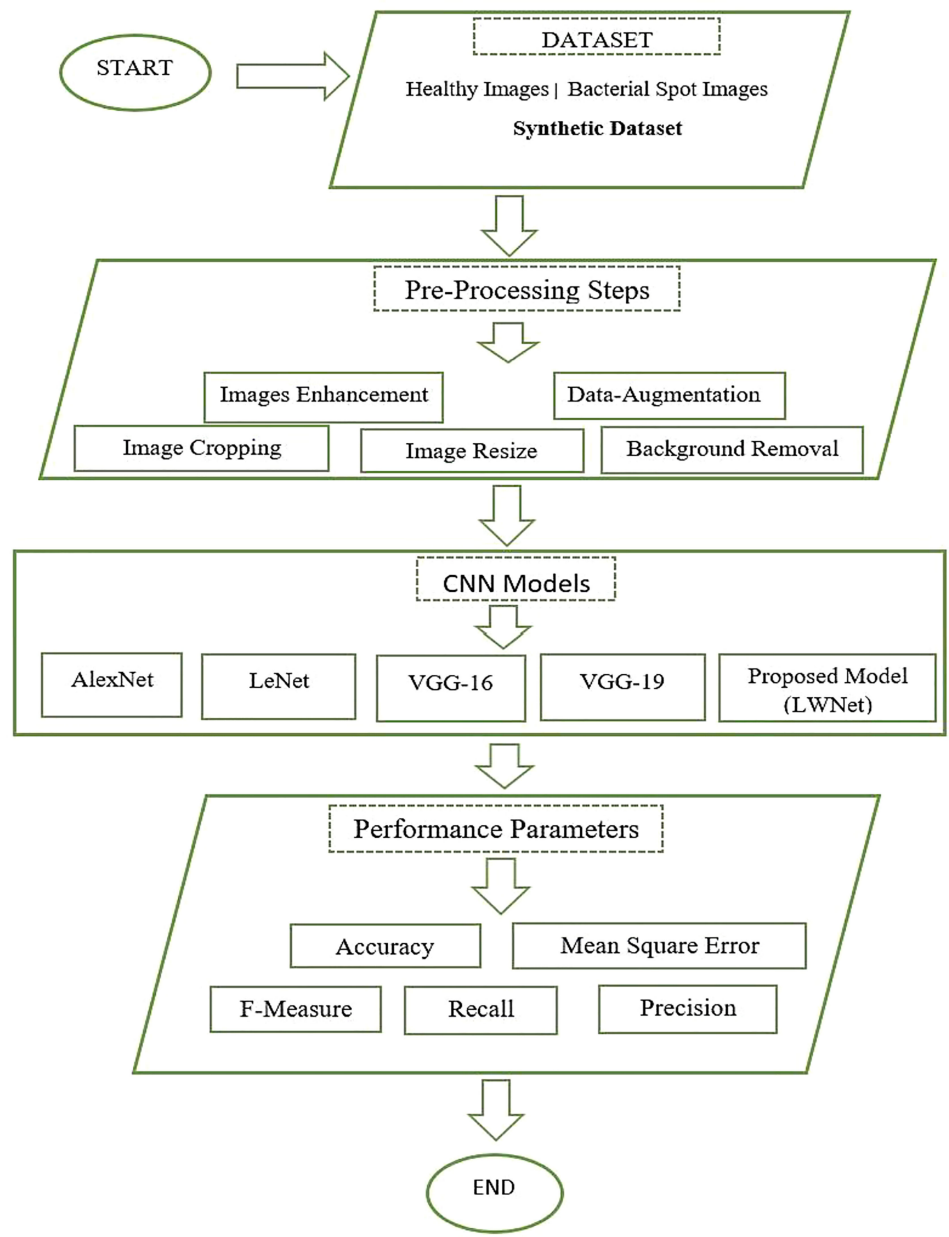
Data flow chart of the proposed LWNet model methodology.

Bacteriosis or Bacterial leaf spot, scientifically also called bacterium Xanthomonas campestris pv. Pruni is a frequent disease affecting nectarines and elderly peach trees. This disease primarily acts on the vulnerable parts like the leaves and fruits of the peach tree and spreads across the entire field. Leaf, flowers, stems, and fruit are commonly used to identify plant diseases. Plant leaves, in particular, are essential to botanists because they have a distinguishing feature. However, manually identifying and recognizing plant diseases is an extremely exhausting and time-consuming operation for the botanist to perform. As a result, an automatic detection system is required to determine Bacteriosis in peach trees, which would benefit botanists and farmers to make good revenue from the field. This paper employs the CNN models to classify Bacteriosis accurately. The suggested method extracts the feature from the images of the peace leaves and classifies them as healthy or infected images using CNN features. **Figure 1** shows the complete methodology adopted to perform this research work. This work starts with collecting the synthetic dataset, followed by the images’ pre-processing. Afterward, CNN models, including our proposed Model (LWNet), are trained and tested. The results collected for all models are evaluated based on the performance parameters. For experimentation of this work, Google Collaboratory, called Google Colab, which provides python based with the cloud GPU and TPU facilities worldwide, is used. Details of each step are discussed in the section below.

### 3.1 Dataset collections

Dataset collection is the essential step of any research work. In this work, we have collected images of the Peach leaves from the research Farm of The Agriculture University Peshawar, Pakistan. The Research Farm comprises around one thousand (1000) Peach trees orchards spread over 14 acres of area. The images were captured through a Canon High-Definition Digital Single-Lens Reflex camera. The size of each image is approximately 5184 X 3456. A total of 625 healthy and 375 infected images were captured from the research farm. Dataset is artificially increased by applying some Data-Augmentation techniques shown in **Table 1**. Data augmentation is a technique in which the researcher artificially increases the dataset to increase the accuracy of machine learning models. The augmentation technique is applied for investigating site datasets like (Rotation, Flipping, Scaling, Brightness, and Translation). In rotation, all the images are rotated at 150 angles. In flipping, all the original images are flipped using horizontal and vertical flipping techniques. In scaling, all the original images were scaled 180%. In brightness, images were brighten using the method iaa.addToBrightness((50)). In translation, the images were translated at TranslationX and TranslationY. The dataset consists of 10000 images, of which five thousand and hundred (5500) are healthy images of peach leaves and four thousand and five hundred (4500) are images of infected leaves having Bacterial spots or Bacterial shot holes. **Figure 1** shows the research flow chart.

**Table 1:** The developed dataset details.

S.No	Types of peach leaves	Original images	Data Augmentation technique	No of images
1	Bacterial Spot	375	rotation, Flipping, Scaling, Brightness, and Translation	4500
2	Healthy Images	625		5500
3	Total	1000		10000

### 3.2 Healthy image

**Figure 2:**
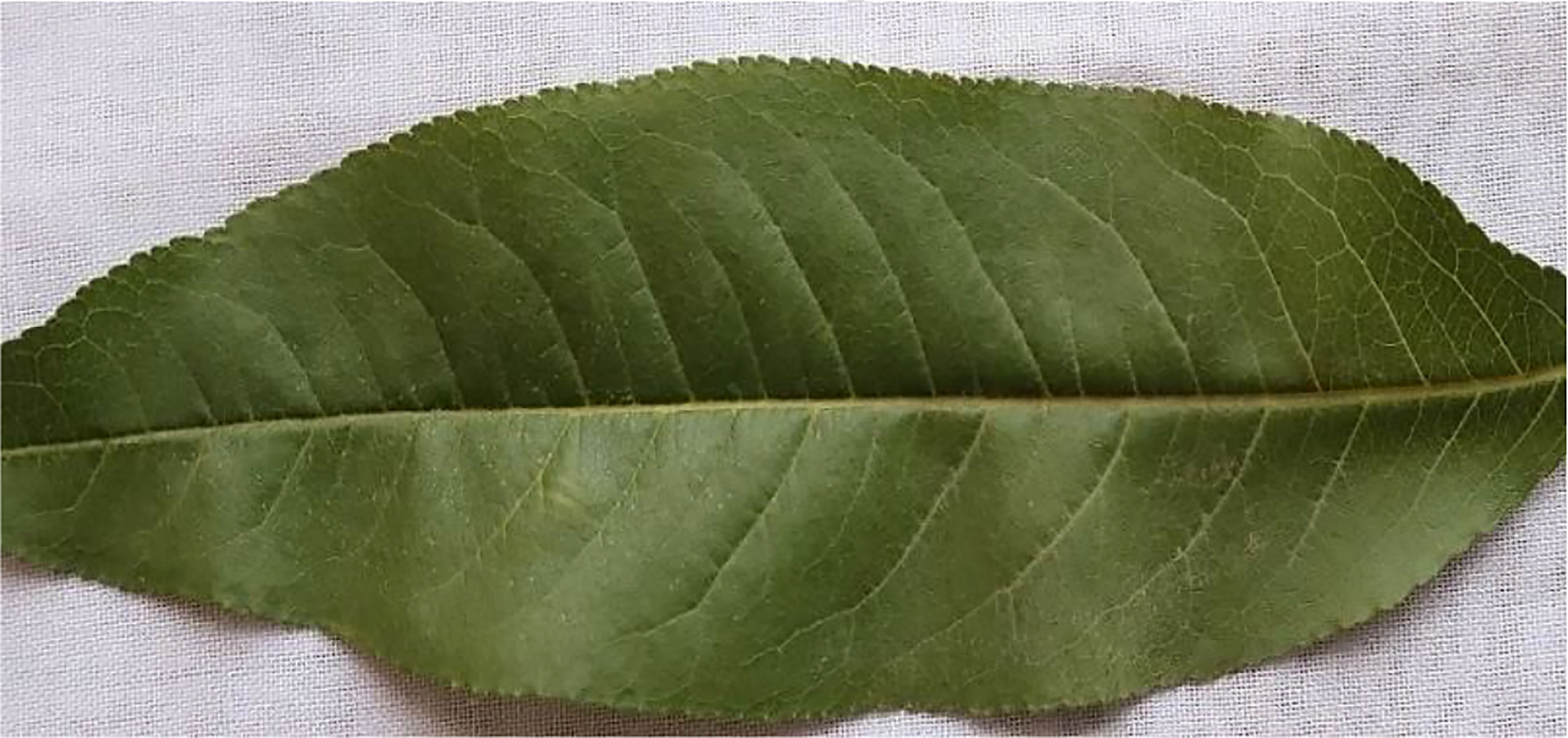
Healthy image of the peach leaf.

**Figure 2** shows a healthy Peach leaf image. They are botanically classified as *Prunus Persica L*. Peach leaves range in size from small to medium and have an oval to lanceolate form. They are typically 10–20 centimeters long and 2–8 centimeters wide. There is a central midrib, and numerous tiny veins branch out from it. Peach leaves are available from spring through summer.

### 3.3 Bacterial spot(leaf)

**Figure 3:**
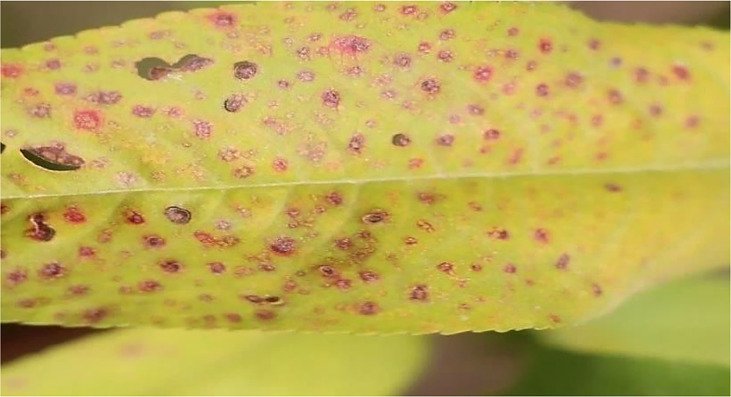
Bacterial spot of the peach leaf.

**Figure 3** shows the Peach leaf with a bacterial spot or shot hole, a widespread condition on peach trees. The bacterium XANTHOMONAS CAMPESTRIS PRUNE causes this leaf spot disease on peach trees.

### 3.4 Pre-processing

Before moving to image analysis, data processing is a crucial stage used to check the data values of an experiment. The image should be prepared to get a decent result. Following are some steps to be conducted for image pre-processing operation, including image resizing, noise removal, image enhancement, background removal, and some augmentation techniques applied to Bacteriosis and healthy leaf images.

#### 3.4.1 Resize images

**Figure 4:**
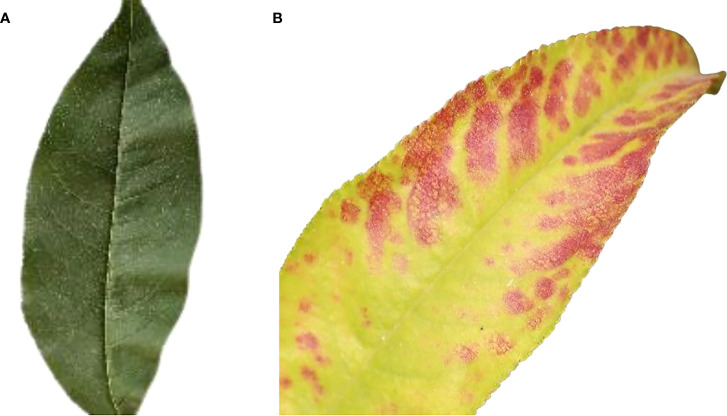
**(A)** Original size of healthy leaf **(B)** original size of bacteriosis leaf.

**Figure 5:**
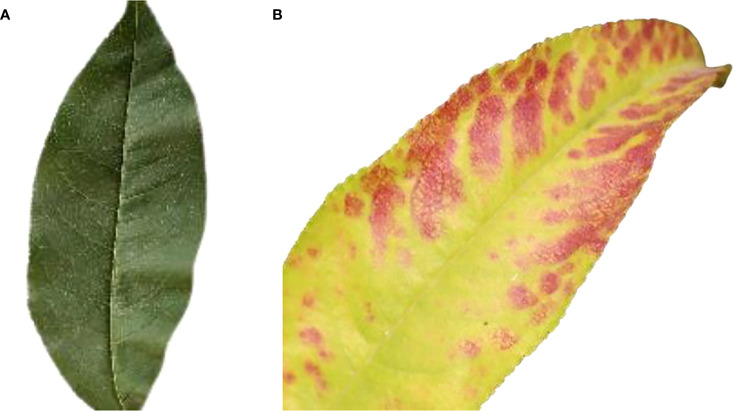
**(A)** Resize image of healthy leaf **(B)** resize image of bacteriosis leaf.

For classification, the dataset contains images of various sizes. As a result, the frame should be resized to a pre-determined size in the early phases. The images would be reduced in size to 227 x 227 pixels. **Figures 4A, B** show the original healthy and infected images before pre-processing whereas **Figures 5A, B** images show the final size of the images used for experimentation. Showed in **Figure 5**.

#### 3.4.2 Noise-removal

The images’ additional information and noise would be removed. So, try some noise cancellation filters or other noise removal techniques such as salt and paper, etc.

#### 3.4.3 Image cropping

When cropping an image, wanted to remove the outer parts of the image that are not interested in.

#### 3.4.4 Image enhancement

**Figure 6:**
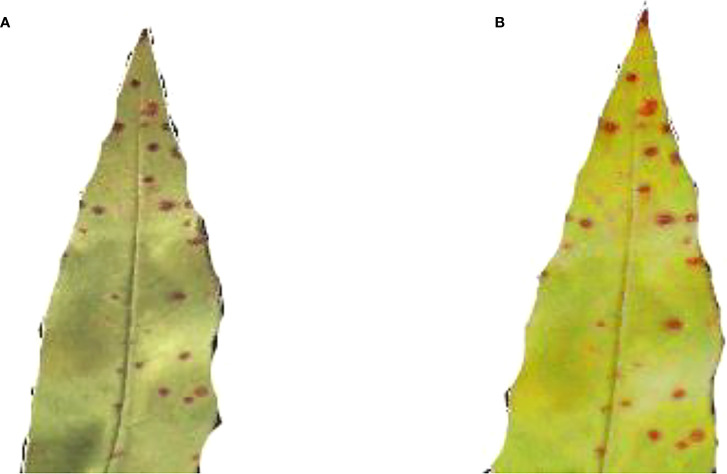
**(A)** Original image **(B)** brighten image.

Image enhancement is the practice of highlighting some information in an image and removing unnecessary information according to our needs like removing noise, revealing blurred, etc. This step makes the image clear for further analysis as shown in **Figure 6**.

#### 3.4.5 Background removal

It is the procedure to erase unwanted items from the image. In our dataset, all the images have a background that could directly affect the experimentation and results. The unwanted background has been removed from the images to improve the results. For this purpose, an openly available software ***remove.bg*,** on Internet has been used.

#### 3.4.6 Feature extraction

Feature extraction is the subsequent stage and is crucial for the classification of the images. Images depict the similarities between things that are inherently related. The classifier utilized functions and labels to compare various photos and group them into distinct classes. Many convolution layers were used in feature extraction, followed by an activation function and max-pooling. CNN characteristics eradicate discriminatory activities (Shafique and Tehsin, 2018).

### 3.5 Activation function

**Figure 7:**
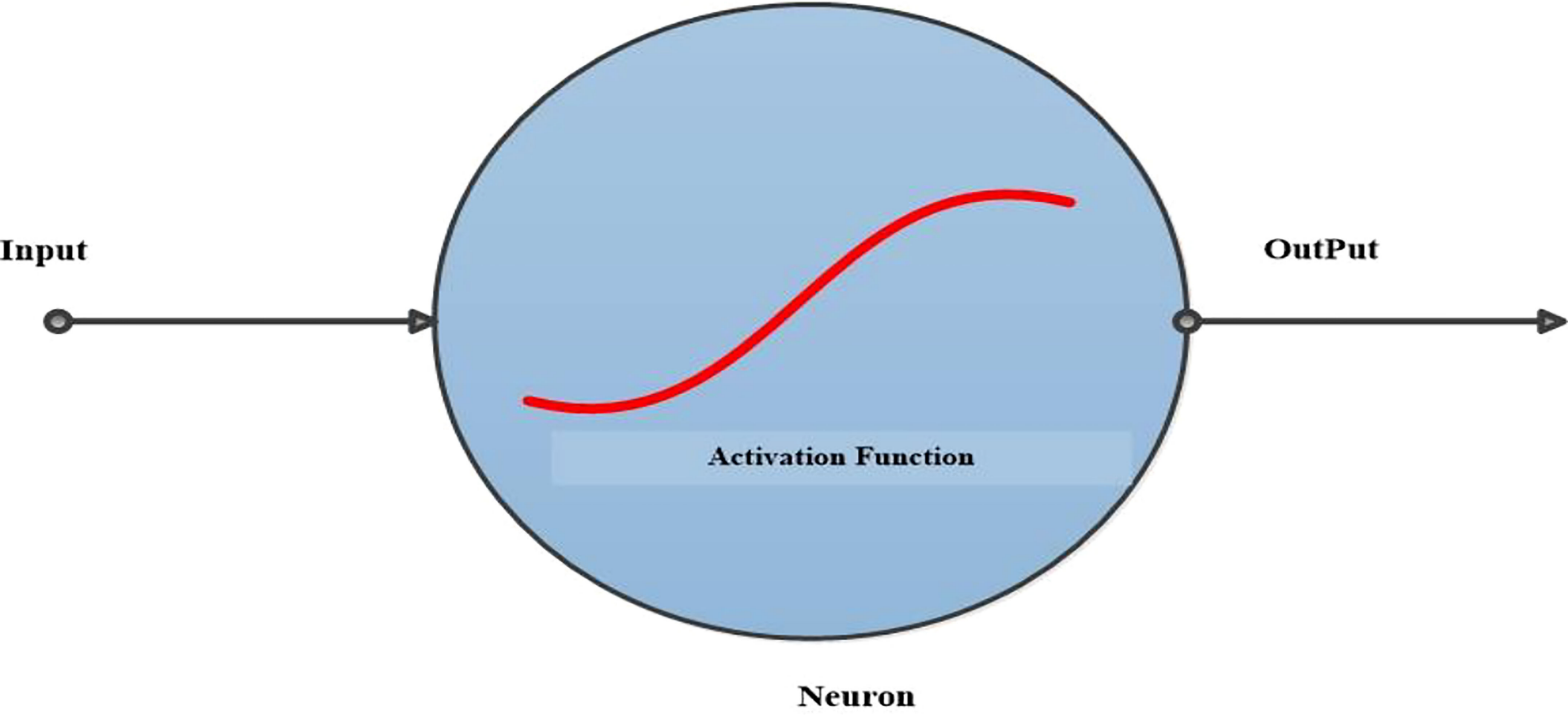
Activation function works.

For neural networks, the activation function is crucial because it determines whether or not a neuron should activate by computing the weight and then adding bias to the result. Performing a non-linear presentation in neuron release is the function’s main goal. As displayed in **Figure 7**.

Non-linear activation functions are increasingly used in neural networks to help the network comprehend complicated input, compute and understand practically any function representing the query, and make exact predictions. There are a variety of activation mechanisms that can be adopted in the neural network. Below is the list of commonly used functions.

1. Sigmoid or Logistic
2. Hyperbolic Tangent Tanh
3. Rectified linear unit ReLu

#### 3.5.1 Sigmoid or logistic

**Figure 8:**
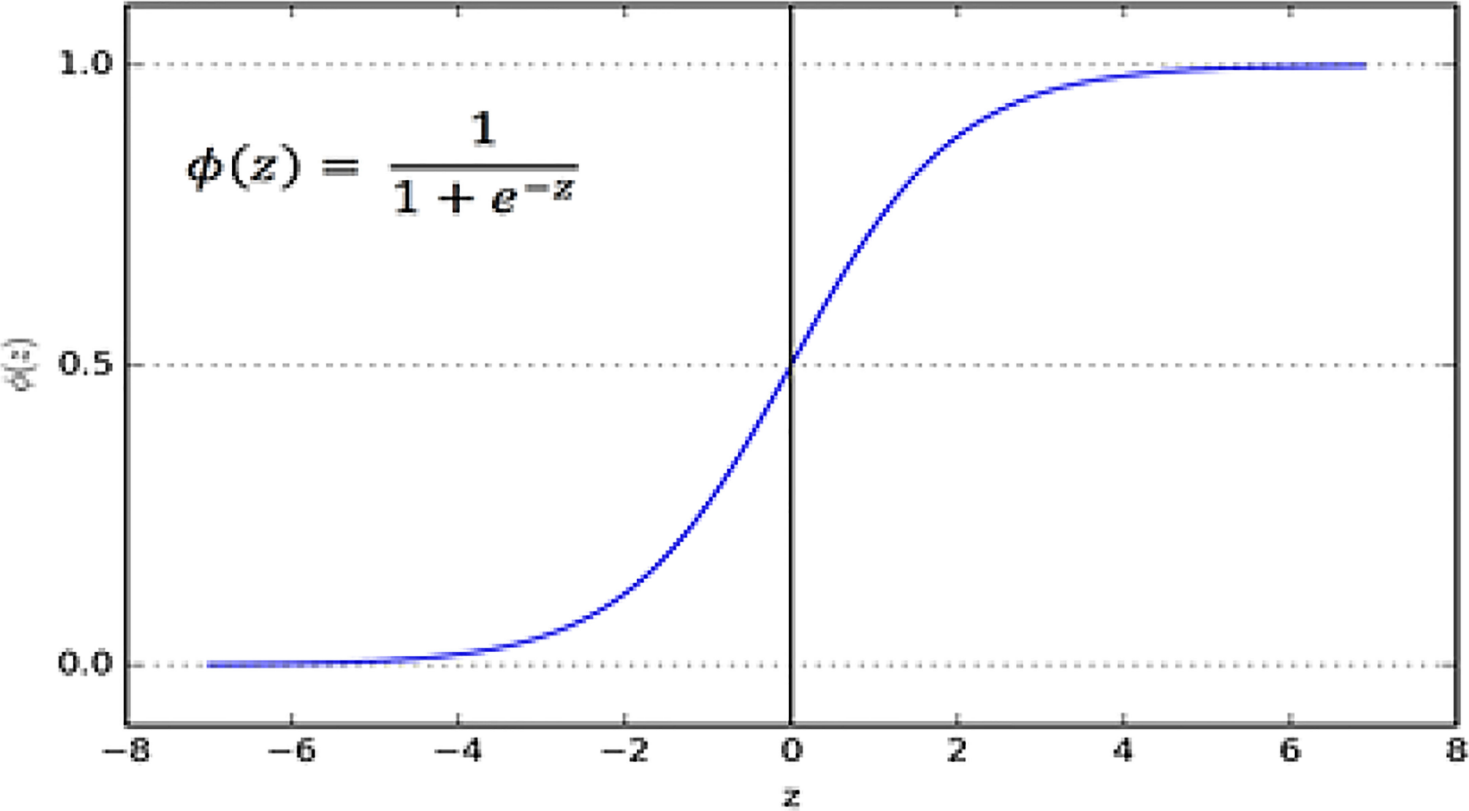
Sigmoid function.

In the Sigmoid activation function, its input and output are actual values. The sigmoid function prevents output value overflow and has numerous advantages over smooth gradients. A precise prediction of X above 2 or less typically places the value of Y very close to 1 or 0 at the edge of the curve shown in **Figure 8**. Such values allow precise predictions.

#### 3.5.2 Hyperbolic tangent Tanh

**Figure 9:**
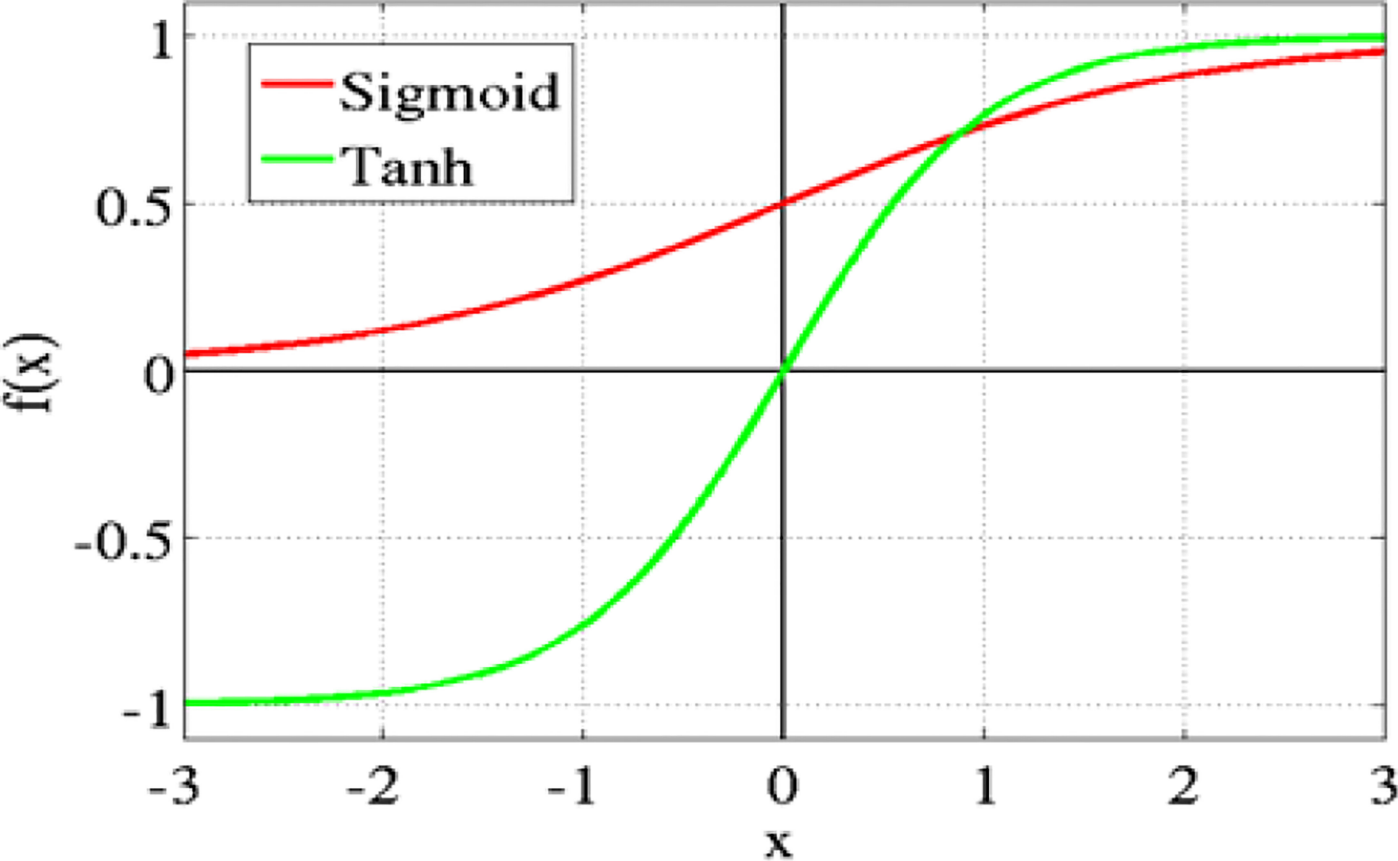
Hyperbolic tangent function.

**Figure 9** shows the Hyperbolic Tangent Tanh function. *It* is an alternative to the sigmoid function and is superior to it. Its value ranges from 1 to -1. The mean for the hidden layer is 0 or very nearly so. This approach makes optimization simpler, although it still uses sigmoid functions.

#### 3.5.3 ReLU activation function

**Figure 10:**
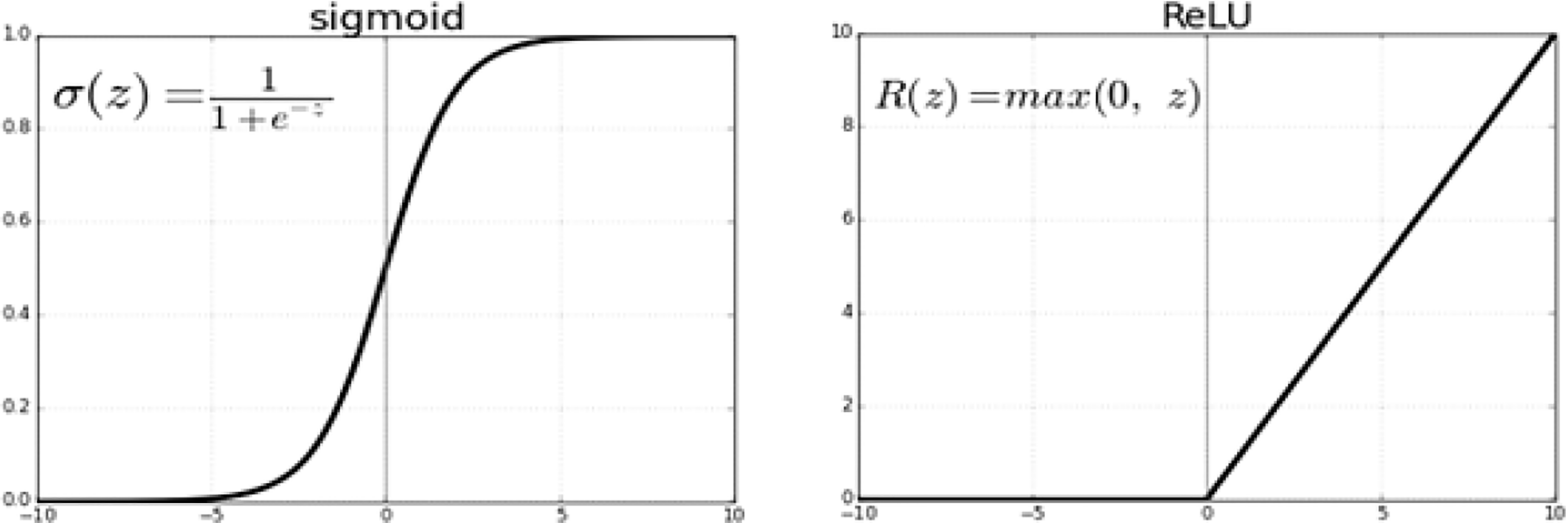
ReLU activation function.

To capture the non-linear dependencies in data that neural networks require, Alexnet, VGG-16, and VGG-19 use rectified linear units. **Figure 10** illustrates this.

ReLU is not saturated, but the sigmoid function is. In terms of training time, this non-linearity saturating is significantly non-saturating non-linearity is slower as the gradient decreases. ReLU worked obtained 25%, which is 6 times the error rate of the tanh function as shown in eq (1).


 (1) Rectified Linear Unit(ReLU):f(x)=max(0,x)

#### 3.5.4 Overfitting problem

The Model successfully reads the training database. It performs well there but poorly on a holdout sample. When the training set’s Accuracy exceeds the validation or testing set, the Model is overfitted, which is why we would try to minimize the issue. Describe an approach for reducing overfitting called data augmentation.

#### 3.5.5 Tuning of hyperparameters

A variety of hyperparameters influences the performance of Deep CNN, including the count of epochs, activation function, concealed layers, dropout, learning rate, nodes, batch size, and others. Experiments are performed with various hidden layers, epochs, activation functions, and learning rates in tuning the parameters. The Model’s ideal state is swiftly attained with the highest Accuracy after fine-tuning.

### 3.6 Proposed CNN model

Researchers have used various pre-trained models for detecting plant diseases. However, it is vital to remember that these models were constructed with many layers to handle cases when the no’s of classes is quite large. If there are more layers, the polynomial being employed in the Model has a higher degree. These require vast quantities of storage and prolonged processing speeds to handle the settings. It should be emphasized that plants often have fewer than 15 different disease classes. Pre-trained models can be overfitting and deliver inaccurate results when applied to such plant data. Similarly, inaccurate results due to underfitting also may arise in a simple linear model without hidden layers. It is therefore suggested to build a more simplified CNN model that is more suitable for a small number of classes. We propose a Lightweight with the following properties.

1. The proposed LWNet model is acquired by compressing VGG-19 layers. The top convolution layers have not detected too good features, or the broad feature compared to the end convolution layers, which detected broad features. We have removed the top three convolution layers, which have fewer filters like 64, 64, and 128, respectively. Also, the size of the four convolution filters increased from 256 to 280. By reducing the convolution layers in the proposed LWNet model, the Accuracy increased to 98.87% from 98.74% when the Model was trained for 50 epochs.
2. In VGG-19, the parameters are quite high. High parameters result in the Model’s overfitting and require much storage. Similarly, low parameters mean less information and underfit Model. An average parameter model is required to get better classification results for a model. The proposed LWNet model is provided in **Table 2**.

**Table 2:** A diagrammatic view of the proposed model is provided.

Layer	Layer type	No of kernels	Kernel size	Output size
1	Convolutional.	128	3×3	128×227×227.
2	Max Pooling.	–	2×2	128×113×113.
3	Convolutional.	280	3×3	280×113×113.
4	Convolutional.	280	3×3	280×113×113.
5	Max Pooling.	–	2×2	280×56×56.
6	Convolutional.	280	3×3	280×56×56.
7	Convolutional.	280	3×3	280×56×56.
8	Max Pooling.	–	2×2	280×28×28.
9	Convolutional.	512	3×3	512×28×28.
10	Convolutional.	512	3×3	512×28×28.
11	Max Pooling.	–	2×2	512×14×14.
12	Convolutional.	512	3×3	512×14×14.
13	Convolutional.	512	3×3	512×14×14.
14	Max Pooling.	–	2×2	512×7×7.
15	Convolutional.	512	3×3	512×7×7.
16	Convolutional.	512	3×3	512×7×7.
17	Max Pooling.	–	2×2	512×3×3.
18	Convolutional.	512	3×3	512×3×3.
19	Convolutional.	512	3×3	512×3×3.
20	Max Pooling.	–	2×2	512×1×1.
21	Fully Connected.	–	–	4096×1×1×1.
22	Dropout	–	–	4096×1×1×1
23	Fully Connected.	–	–	4096×1×1×1.
24	Dropout	–	–	4096×1×1×1
25	Connected with Sigmoid	–	–	2×1×1×1.

**Table 2** explains the structure of the proposed LWNet Model. It has 23 layers altogether 13 convolutional layers, 7 Max Pooling layers, and 3 Dense layers. Only 13 are weight layers, also referred to as learnable parameters layers. The LWNet has an input tensor had three RGB channels and a size of 227, 227. The most notable aspect of the LWNet Model is that it continuously used the same padding and max pool layer of a 2x2 filter with stride 2 and prioritized convolution layers of a 3x3 filter with stride 1 over a huge number of hyper-parameters. The convolution and max pool layers are uniformly placed across the whole architecture. The Conv-1 Layer consists of 128 filters, the Conv-2,3,4 and 5 Layers of 280 filters, the Conv-6,7,8,9,10,11,12 and 13 Layer of 512 filters, Three Fully Connected (FC) layers, the third of which performs 1000-way of classification and contains 1000 channels, are added after a layer of convolutional layers. Each top two FC layer has 4096 channels (one for each class). The final layer was the Sigmoid layer.

**Table 3** shows the parameters of the proposed LWNet model. The dataset consists of 1000 images, of which 70% are used for training and 30% for testing the Models. The LWNet Model uses 13 convolutional layers, the count of max-pooling is 7, and the dropout rate is 0.5 with the ReLu activation function. The LWNet Model is trained for 50 epochs. The number of epochs remained the same for each Model.

**Table 3:** The hyperparameters used in the proposed model.

Hyperparameter	Description
No. of convolution layers	13
No. of max pooling layers	07
Drop out rate	0.5
Activation function	Relu
Learning rate	0.001
No. of epoch	50
Batch size	32
Training size	70%
Test size	30%

The training and testing datasets will be separated from the primary datasets. The training dataset contained 70% (7000 images) of the data from the primary dataset, while the remaining 30% (3000 images) of the dataset has been utilized for testing. A 70% image-based training dataset was utilized for training the Model. A 30% image-based testing dataset was used to test the Model.

#### 3.6.1 Simulation parameters

Experiments are performed on images of healthy leaves and infected (Bacteriocins) leaves collected from the research Farm of Agriculture University Peshawar, Pakistan. “Colab is an online platform based on python employed to carry out a structured program. The research used the deep state-of-the-art of CNNs using LeNet, Alexnet, VGG-16, and VGG-19 and proposed the LWNet Model. Experimental findings with a 98.87% accuracy rate are presented. Furthermore, to classify the bacterium XANTHOMONAS CAMPESTRIS PRUNE illness in real-time. As discussed in section 3.1, the dataset consists of 1000 images, of which 70% were used for training and 30% for testing the Model. The total number of epochs remains constant for each Model. Each Model has its activation function. It is also the same for each Model before the sigmoid function’s final performance. The parameters were set for experiments, as shown in **Table 4**, which showed the structure of the proposed LWNet model.

**Table 4:** Simulation parameters of LWNet model.

Name’s	Parameter’s
Algorithm	Proposed Model Based on VGG-19
Convolution-Layers	ReLU Activation Function
Fully Connected-Layers	Sigmoid Activation Function
Maximum Number of Epochs	50
Dataset	10000 images
Data for Trained the Model	70(%)
Data for testing the Model	30(%)
Environment	Google Colab
Evaluation-Parameter	Accuracy, MSE Loss, Precision, Recall, and F-Measure

**Table 4** shows the simulation parameters used for the experimentation of the proposed LWNet model, including the training and testing processes. The LWNet uses Relu activation function to increase non-linearity. As discussed earlier, the simulation of each Model is performed for 50 epochs, and the Sigmoid function is used for classifying images into healthy and infected classes. These simulations are performed on Google Colab. Each Model’s results are evaluated using Accuracy, MSE loss, precision, Recall, and F-Measure.

#### 3.6.2 Evaluation parameters

The model performance was evaluated by using the performance parameter. Mean Square Error (MSE) loss, Accuracy, Precision, Recall, and F-measure parameters have been employed to assess the LWNet Model and compared with other CNN models. Equation (2) computes the Accuracy of the classification. All correct measurements were calculated with Accuracy. The accurate prediction is divisible by the total number of observations.” The system’s performance would be good if the classification accuracy is high. Equation (3) represents the precision, Equation (4) shows the Recall, and Equation (5) denotes the F-measure.


(2) $$\text{Accuracy}=\frac{\left(\text{TrP}+\text{TrN}\right)}{\left(\text{TrP}+\text{TrN}+\text{FsP}+\text{FsN}\right)}$$

(3) $$\mathrm{Pr}ecision=\frac{TrP}{TrP+FsP}$$

(4) $$Recall=\frac{TrP}{TrP+FsN}$$

(5) $$F-Measure=2\frac{precision*recall}{\;precision+recall}$$

whereas TrP represents True Positive, TrN denotes True Negative, FsP depicts False Positive, and FsN represents False Negative.

## 4 Experimental results

Experiments were performed on bacteriosis and healthy images were collected from the research Farm of Agriculture University Peshawar, Pakistan. “Colab is an online platform based on python that was used to run a structured program. This study used the deep state of the art of convolutional neural network “ Moreover, to classify bacteriosis images into healthy images using LeNet, Alexnet, VGG16, VGG-19, and proposed model based on VGG-19. Experimental results are presented and achieved an accuracy of 98.87%. Furthermore, to classify the bacterium XANTHOMONAS CAMPESTRIS PRUNE disease in real-time. From the dataset. The total number of images in our dataset was Ten Thousand of which 70% of images were used for training and 30% for testing.

For each model, the total number of epochs remains constant. Each model has its activation function. And before the ultimate performance of the sigmoid function, it is the same for each model.

**Figure 11:**
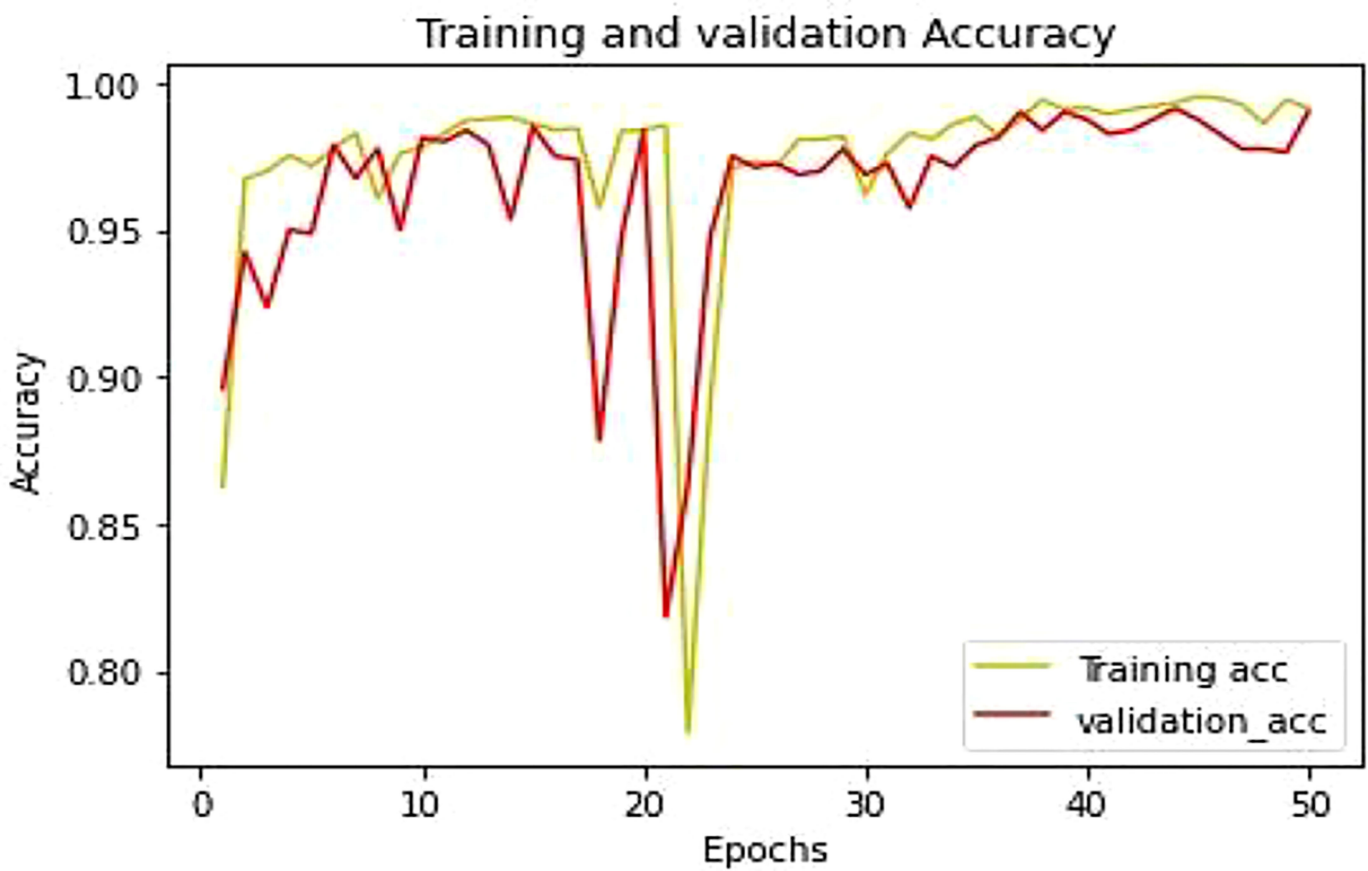
Training and validation models accuracy.

This section described the experimentation results of the proposed LWNet Model and compared them with AlexNet, LeNet, VGG-16, and VGG-19. **Figure 10** shows the accuracy of the proposed LWNet for training and validation. The training accuracy starts at 86% at the first epoch of the simulation and achieves 98.87% by 50 epochs. Similarly, the accuracy of the validations begins at 90% at the first epoch and attains 98.87% at the 50 epochs. **Figure 11** shows the training and validation loss of the LWNet. The results depict the initial value of the loss as 0.2 and reach 0.0113 for both training and validation of the system.

### 4.1 Parameters of the proposed model

For experiments following parameters were set as shown in **Table 5** showed the structure of the proposed model.

**Table 5:** Parameters Detail of Proposed Model.

Name	Parameters
Algorithm	Proposed Model Based on VGG-19
Convolution Layers	ReLU Activation Function
Fully Connected Layers	Sigmoid Activation Function
Maximum Number of Epochs	50
Data Set	10000 images
Data for Training	70%
Data for testing	30%
Environments	Google Colab
Evaluation Parameter	Accuracy, MSE Loss, Precision, Recall, and F-Measure

**Figure 12:**
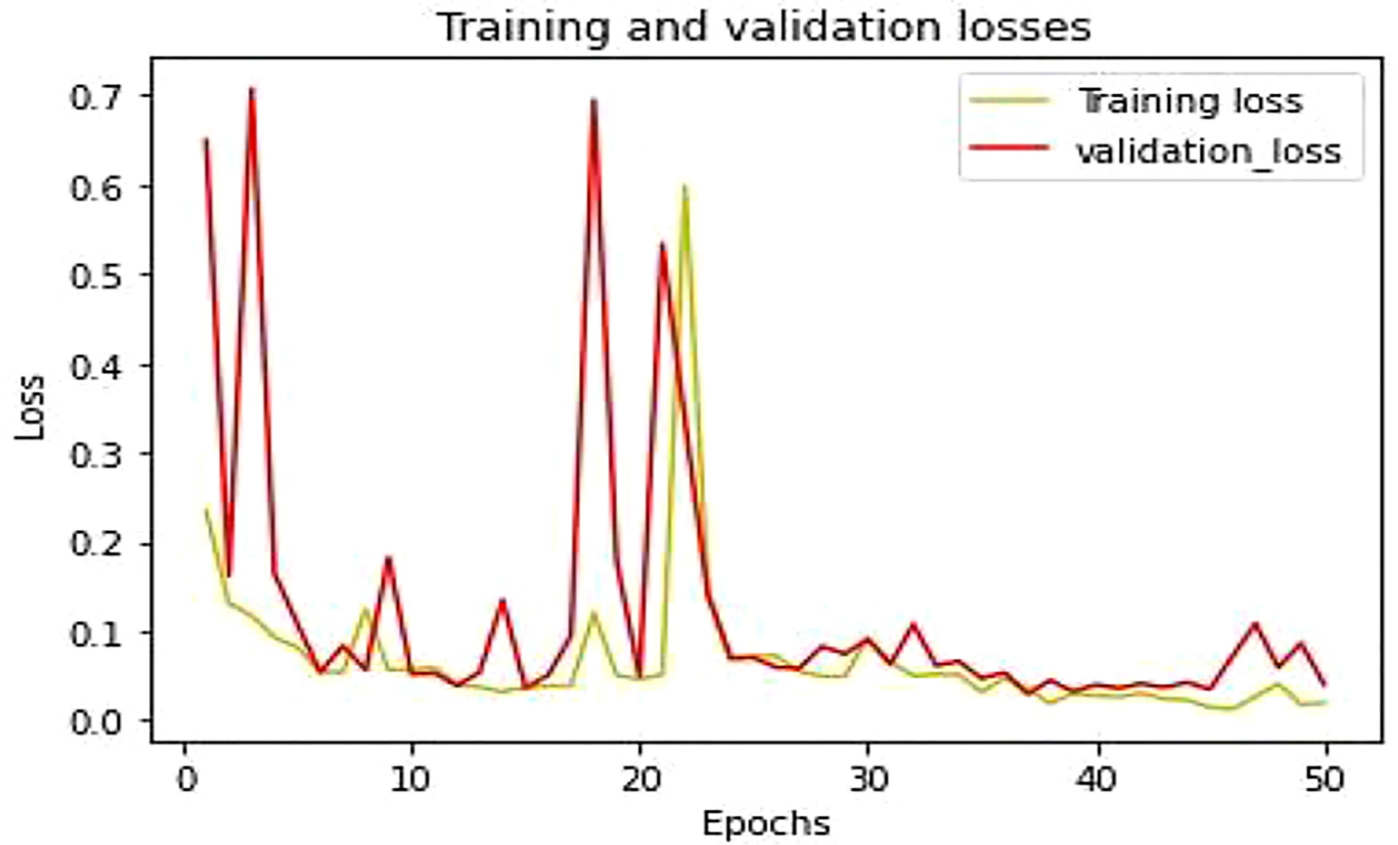
Training and validation model loss.

**Table 5** shows the parameter of the proposed model and how the training and testing processes work. In the first step, all the images were found in the directory. The proposed model contains different layers. The last layer of connectivity was linked with the sigmoid activation function. The last section trained and validated the proposed model that differentiates between the infected leaf and the normal leaf. **Figure 11** showed the model training and testing accuracy while **Figure 12** showed the model training and validation loss

**Table 6** The testing data, which included precision, recall, and F-Measure, were evaluated using the Confusion Matrix True Positive (TP), True Negative (TN), False Positive (FP), and False Negative (FN) values.

**Table 6:** Proposed Model Performance Parameter.

	Precision	Recall	F_measure
*Healthy*	*0.95*	*0.98*	*0.96*
*Bacteriosis*	*1.00*	*0.99*	*0.99*

**Figure 13:**
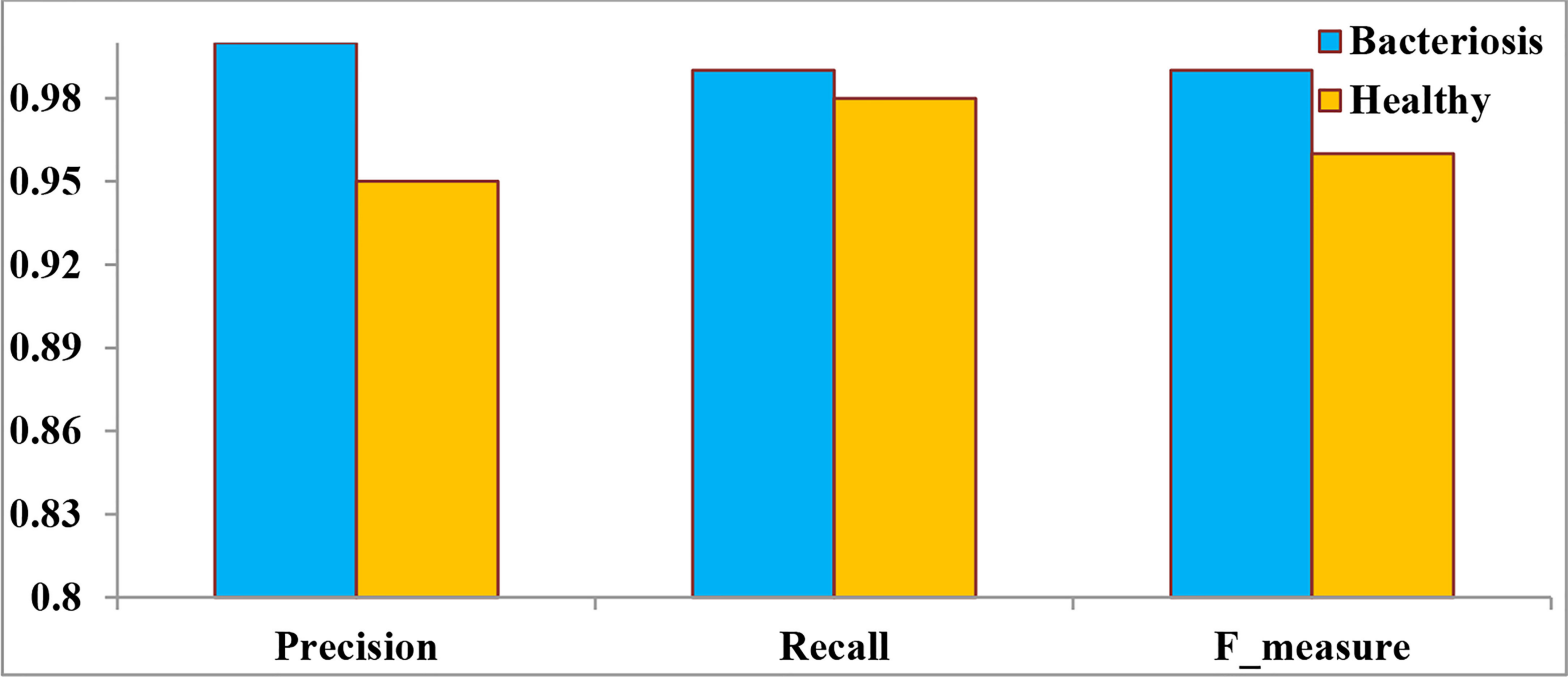
Performance parameter of the LWNet model.

**Figure 13** denotes the performance of the proposed LWNet Model for both healthy and Bacteriosis (infected) leaves. The LWNet achieves a precision of 95% for healthy leaves and 100% for the leaves infected with Bacteriosis. Similarly, the Recall for the classification of healthy leaves is 98% and 99% for the infected leaves. Furthermore, F-measure for the healthy leaves is 96% and 99% for the infected leaves.

**Figure 14:**
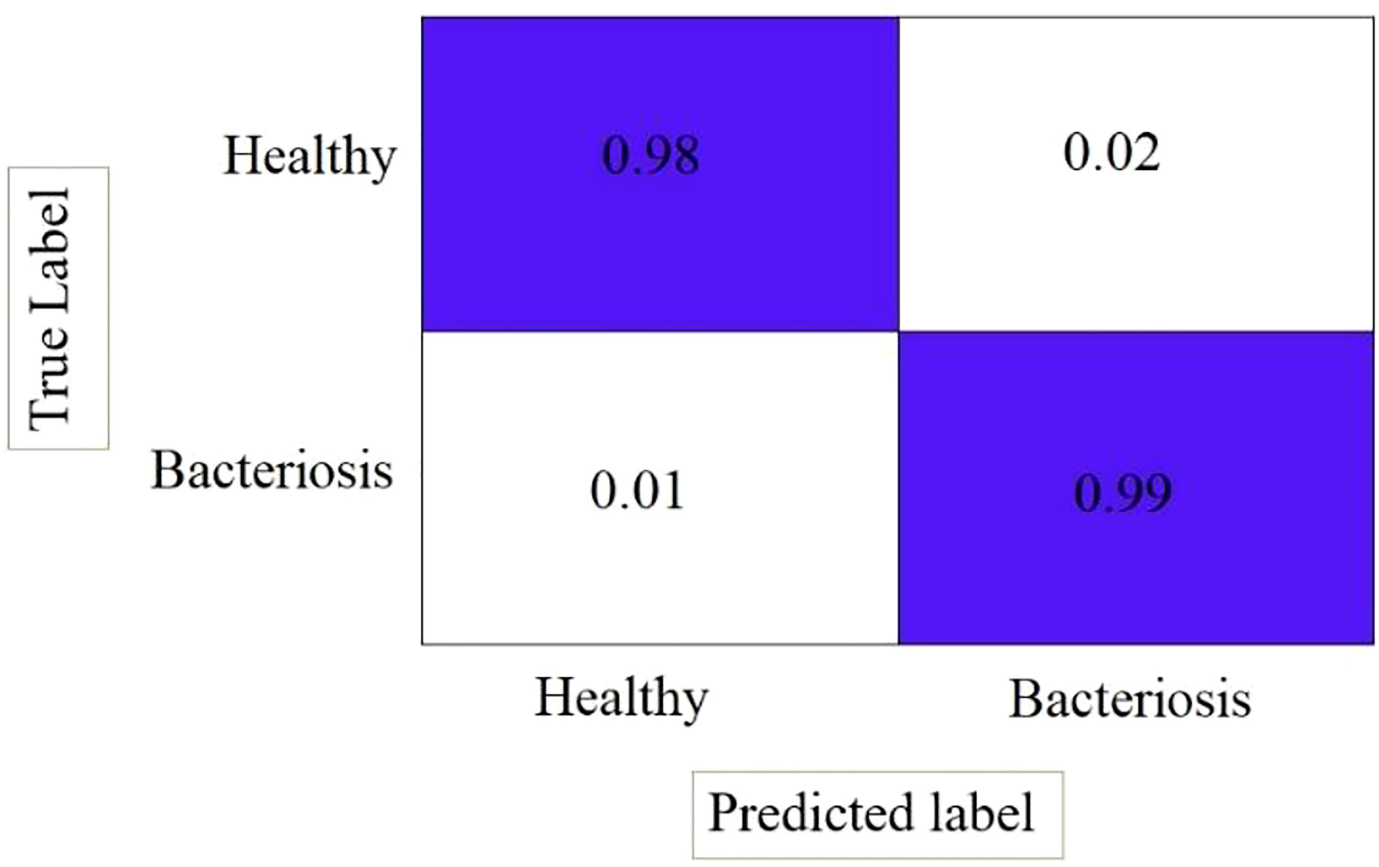
Confusion matrix.

**Figure 14** shows the confusion matrix of the LWNet results, describing that the proposed LWNet Model returns 98% True positive for both healthy and infected leaves and 99% True negative results. The LWNet shows 2% false positives and 1% False negatives.

### 4.2 Accuracy comparison with existing models

**Figure 15:**
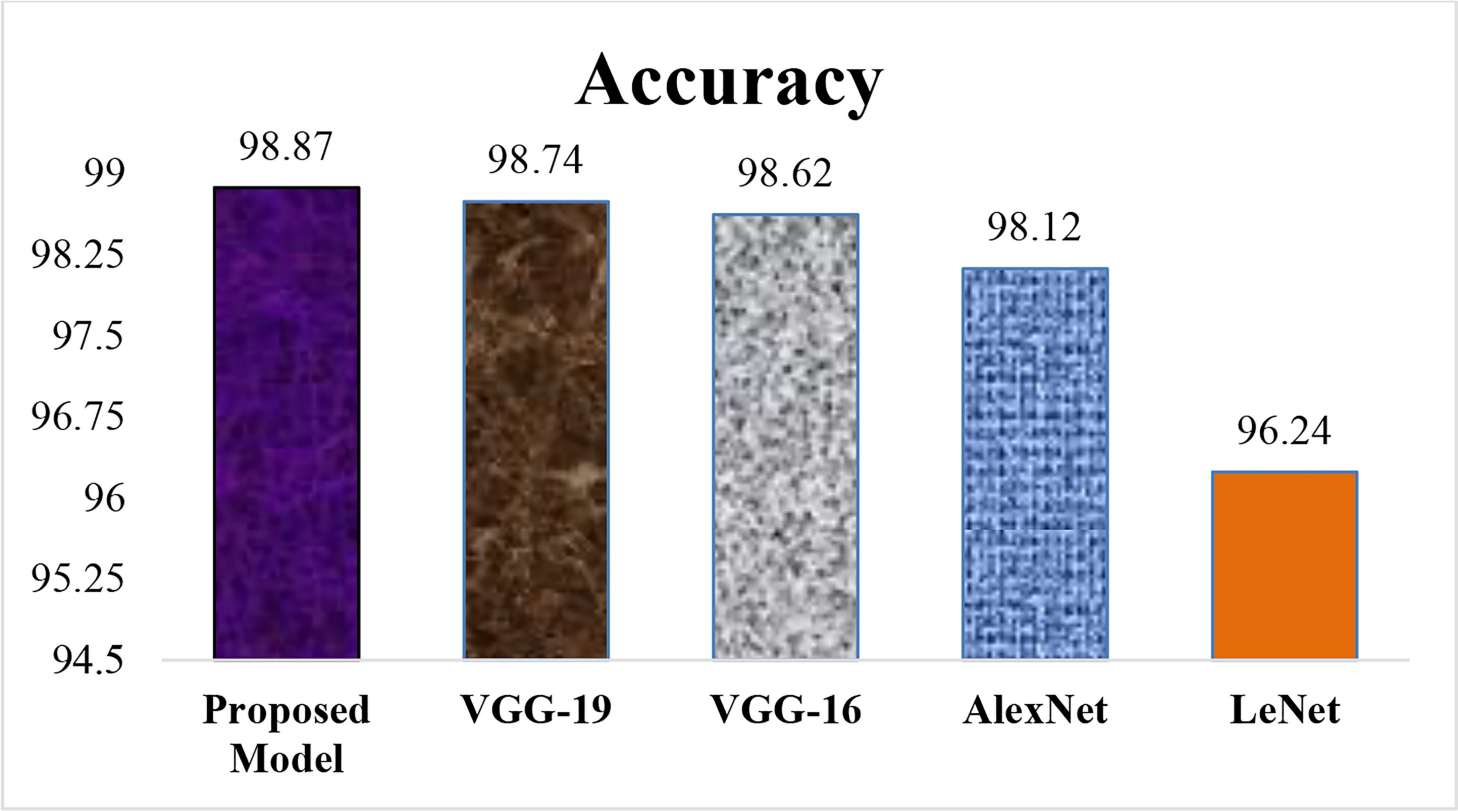
Accuracy (%) of different models.

**Figure 15** describes the Accuracy of the proposed LWNet model and other CNN models. The results show that the LWNet achieves 98.87% accuracy, whereas VGG-19 displayed 98.74% accuracy. Similarly, VGG-16 achieved 98.62% accuracy, AlexNet succeeded with 98.12% accuracy, and 96.24% for LeNet. The proposed LWNet achieves 0.1315% better Accuracy than VGG-19, 0.253% improved results compared to VGG-16, 0.76% better than AlexNet, and 2.63% enhanced Accuracy compared to LENET. as shown in **Table 7** below.

**Table 7:** Accuracy Comparison with existing models.

Model	Accuracy (%)
LeNet	96.24%
Alex Net	98.12%
VGG-16	98.62%
VGG-19	98.74%
Proposed Model	98.87%

**Figure 16:**
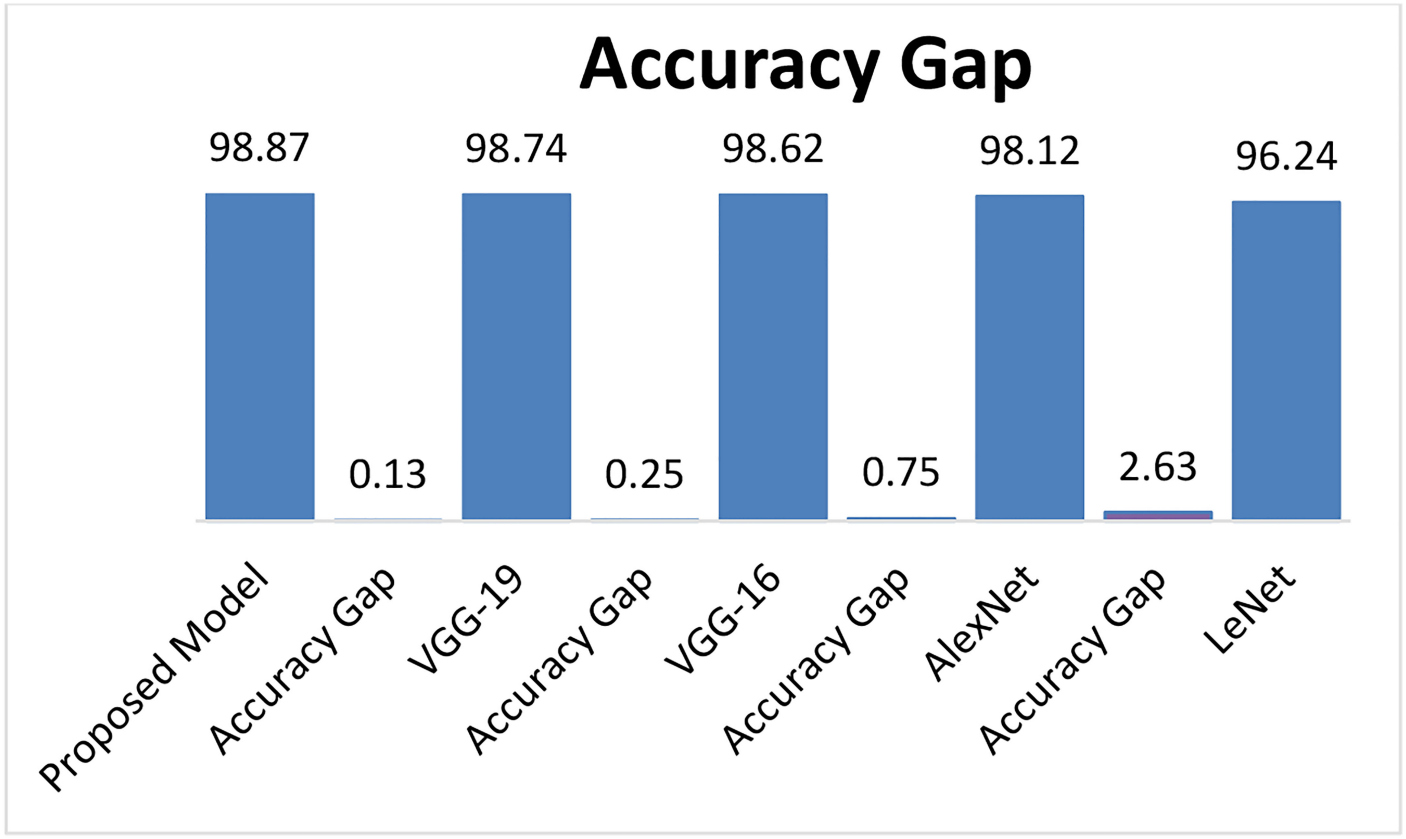
Accuracy gap of different models.

**Table 7** indicated that the proposed model achieved the highest accuracy comparison with LeNet, AlexNet, VGG-16, and VGG-19, models on 50 epochs and classify the two classes as one of the given images of Bacteriosis and healthy. Furthermore, **Figures 15**, **16** showed a graphical representation of the accuracy convergence of the two models.

### 4.3 The comparison of the proposed model loss with other models

**Figure 17:**
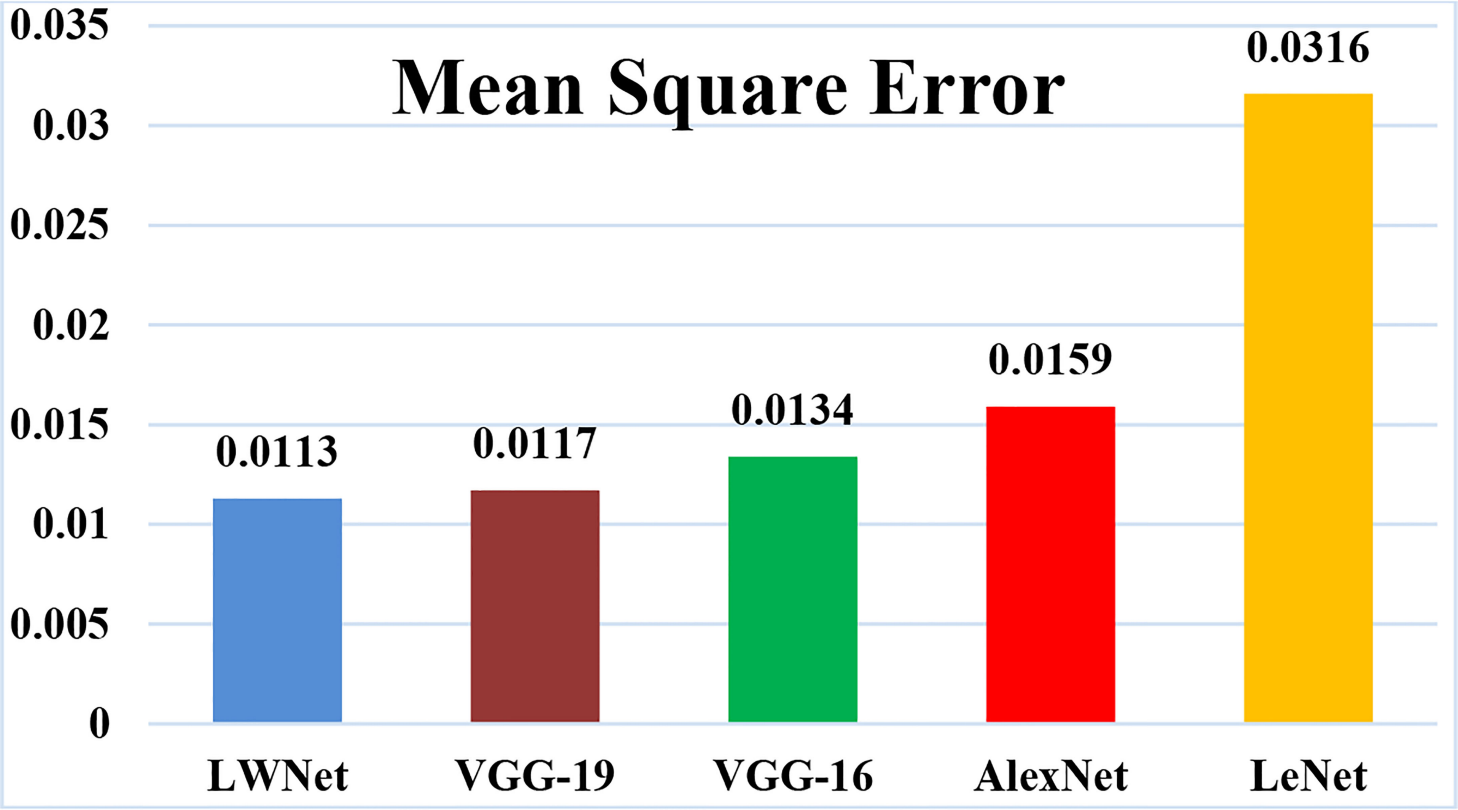
Mean square error of the simulation results.

The mean square error (MSE) loss function is the discrepancy between the anticipated and real values. The LWNet Model experienced the lowest loss of 0.0113 by 50 epochs, shown in **Figure 17**. The LeNet model displayed a 0.0316 loss. The loss gap between LeNet and LWNet Models was 0.0203. Similarly, The AlexNet model loss was 0.0159. The loss gap between AlexNet and LWNet Models is 0.0046%. Also, the MSE for the VGG-16 Model is 0.0134, with a loss gap of 0.0021 compared to the LWNet. Furthermore, the MSE for the VGG-19 model is 0.0117. The loss gap between VGG-19 and Proposed Model was 0.0004%. LWNet achieved low MSE because of the Model’s fine-tuning, including learning rate, layers, filters, and drop out.

The proposed model obtained a loss is 0.0113 as shown in **Table 8**, which is the lowest loss as compared to other CNN models.

**Table 8:** Loss evaluation of different Models.

Model	Losses
Proposed Model	0.0113
VGG-19	0.0117
VGG-16	0.0134
AlexNet	0.0159
LeNet	0.0316

### 4.4 Comparison of simulation time

**Table 9** represents the simulation time of each Model. The total simulation time for the LeNet model is 10 minutes and 20 seconds. As the LeNet Model has fewer numbers of layers with a smaller number of filters size. Similarly, the training and testing time for the ALexNet Model is 14 minutes and 35 seconds. It has the same reasons as LeNet for such a short execution time. VGG-16 finished their training and testing in 1hrs 53 minutes and 15 seconds. VGG-19 completed the simulation in 2hrs 3 minutes and 59 seconds. The running time for the LWNet Model is 1hrs 56 minutes and 38 seconds. LWNet took a shorter simulation time than VGG-19 because the number of layers was reduced from 19 to 16. The dropout rate and batch size also contributed to the shorter execution time.

**Table 9:** Simulation time of Model Execution.

S.No	Models	Hours	Minutes	Seconds
1	LeNet	–	10	20
2	AlexNet	–	14	35
3	VGG-16	1	53	15
4	VGG-19	2	3	59
5	LWNet	1	56	38

## 5 Conclusions

In this research work, the lightweight CNN model has been proposed based on VGG19 for classifying disease in peach leaves. The samples of images of peach leaves for this research were collected from research Farm of Agriculture University Peshawar, Pakistan. The dataset consists of 10000 images Different Augmentation techniques were applied to increase the dataset artificially. Different CNN models like LeNet, Alex net, VGG-16, and VGG-19 were used for the dataset. The act of the LWNet modal was compared with the different evaluation metrics like Accuracy, Precision, Recall, F-Measure, Confusion Matrix, and MSE. The LWNet Model outperformed state-of-the-art Accuracy in leaf-based categorization with an accuracy of 98.87%, which is high among the models. The results of this study might be used to classify images from CT scans of the brain, X-rays of the lungs, liver, or kidneys, and other biological domains to diagnose diseases quickly and cheaply. They could also be applied to other crops besides peach leaves.

The limitations of the study are as follows. The PNG images are only used for Bacteriosis and healthy image detection and classification. The Bacteriosis disease is only done in this study, no other detection tasks are performed.

The objective of a prospective future project would be to gauge the disease’s severity in peach leaves. The proposed work can be expanded to show how widely the disease has gone throughout the plant. The intensity of the illness required at any particular stage of peach plant vegetative and reproductive growth may be determined with this research.

## Data availability statement

The original contributions presented in the study are included in the article/supplementary material. Further inquiries can be directed to the corresponding authors.

## Author contributions

MA conceptualized this study, conducted experiments, wrote the original draft, and revised the manuscript. BS, MH, RUK, TH, FarA, and FA wrote the manuscript, made the experimental plan, performed the experiments, performed the data analysis, supervised the work, and revised the manuscript. IS evaluated the developed technique and revised the manuscript. KSK designed the experimental plan, supervised the work, and revised the manuscript. All authors have read and agreed to the published version of the manuscript.

## Funding

This work was supported by National Research Foundation of Korea-Grant funded by the Korean Government (MSIT)-NRF-2020R1A2B5B02002478). This research work was also supported by the Cluster grant R20143 of Zayed University, UAE.

## Acknowledgments

We thank all the authors for their contribution.

## Conflict of interest

The authors declare that the research was conducted in the absence of any commercial or financial relationships that could be construed as a potential conflict of interest.

## Publisher’s note

All claims expressed in this article are solely those of the authors and do not necessarily represent those of their affiliated organizations, or those of the publisher, the editors and the reviewers. Any product that may be evaluated in this article, or claim that may be made by its manufacturer, is not guaranteed or endorsed by the publisher.
